# Design of Skin Penetration Enhancers Using Replacement Methods for the Selection of the Molecular Descriptors

**DOI:** 10.3390/pharmaceutics4030343

**Published:** 2012-07-11

**Authors:** Laurent Simon, Beshoy Abdelmalek

**Affiliations:** Otto H. York Department of Chemical, Biological and Pharmaceutical Engineering, New Jersey Institute of Technology, Newark NJ 07102, USA; Email: bha5@njit.edu

**Keywords:** skin penetration enhancer, replacement methods, neural networks

## Abstract

Transdermal delivery of certain drugs is challenging because of skin barrier resistance. This study focuses on the implementation of feature-selection algorithms to design chemical penetration enhancers. A database, consisting of 145 polar and nonpolar chemicals, was chosen for the investigation. Replacement, enhanced replacement and stepwise algorithms were applied to identify relevant structural properties of these compounds. The descriptors were calculated using Molecular Modeling Pro™ Plus. Based on the coefficient of determination, the replacement methods outperformed the stepwise approach in selecting the features that best correlated with the flux enhancement ratio. An artificial neural network model was built to map a subset of descriptors from sixty-one nonpolar enhancers onto the output vector. The *R*^2^ value improved from 0.68, for a linear model, to 0.74, which shows that the improved framework might be effective in the design of compounds with user-defined properties.

## 1. Introduction

Transdermal drug delivery is the transport of a therapeutic chemical substance into the body through the skin in the forms of ointment or a patch. The first transdermal drug-delivery system (TDDS), dating back to 1979, consisted of a scopolamine patch prescribed to treat motion sickness [1]. However, until the release of nicotine patches in 1991 [1], little attention was paid to this approach. Currently, there exists over 21 TDDSs. They are becoming more accepted, by the pharmaceutical industry, as viable drug-delivery methods.

The dermal route offers several advantages. Applying a transdermal patch is easy, painless and rapidly effective, as opposed to hypodermic injections or oraldrug-delivery systems, which usually require a long time for the medication to reach its target site. In spite of these benefits, only a limited number of drugs are administered using this method because of the skin barrier properties. The dermal membrane consists of three main layers of tissue: the epidermis (stratum corneum and viable epidermis), dermis and a subcutaneous region [2]. The skin is particularly resistant to the permeation of most external chemicals due to the presence of the stratum corneum, composed mainly of dead cells.

Chemical penetration enhancers (CPEs) are designed to stimulate transport across the skin. These sorption promoters (or accelerants) increase the drug flux and reduce the resistance of the skin barrier [3]. Penetration enhancers are added to the formulation of TDDSs to allow the permeation of molecules that would have crossed the dermal layer at a rate too slow for clinical applications or have been precluded from traversing the biological membrane. The effectiveness of an accelerant is measured by the Enhancement Ratio (ER), defined as the ratio of the flux in the presence of a fixed concentration of CPE to the delivery rate when the enhancer is not added to the formulation [4]. The application of CPEs has had positive impact on TDDSs. As a result, efforts have been devoted to the development of several numerical tools to help design CPEs that exhibit desired properties. 

Previous studies suggest that the activity of a penetration enhancer is related to the structure of the drug as well as that of the enhancer [5]. A quantitative structure-activity relationship (QSAR) modeling framework can be adopted to derive mathematical relationships between the ER and the enhancers’ molecular descriptors (or features) [4]. However, the number and type of features to include in a model is generally not known *a priori*. In this study, three different methods for determining an optimal subset of molecular descriptors are investigated: the replacement method (RM), the enhanced replacement method (ERM) and the traditional forward stepwise regression. Artificial neural networks (ANNs) are then implemented to produce more accurate models. This contribution is the first to employ ERM and RM in selecting the best descriptors for transdermal applications.

The RM and ERM are provided to help identify the best combination of descriptors from a large pool of variables. Artificial neural networks are built to map a series of input patterns onto a set of output variables. When possible, these ANN models can be implemented to refine the initial QSAR models.

### 1.1. Replacement Method for the Selection of QSAR Descriptors

Given a multiple linear regression model:

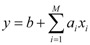
(1)
where *x_i_* is a feature (e.g., molecular weight) from a pool of size *N* and *y* is the output variable. The replacement method (RM) helps select a subset of *M* descriptors that result in the lowest standard deviation from Equation 1. In this procedure, an initial set of *M* features (*V*) is selected arbitrarily [6] from a population *W*. One of the descriptors in *V* is chosen (first path) and replaced one at a time by the remaining descriptors from *W − V*. The set that yields the smallest standard deviation (*S*) is saved [7]. The second step involves choosing the descriptor with the coefficient showing the greatest relative error and replacing it with features from the remaining set. Note that the replacement is made if it decreases *S*. This scheme is repeated until the set of descriptors remains unchanged. For each cycle, the descriptor optimized in the preceding cycle is not altered [6,8]. In the end, the best descriptor of the first path is obtained. The procedure continues for all of the *M* possible paths and the one with the smallest *S* is retained. 

### 1.2. Enhanced Replacement Method for the Selection of QSAR Descriptors

The ERM can be applied to predict an optimal group of molecular descriptors from a large set. This technique consists of the implementation of two algorithms, the RM and a modified RM (or MRM), in the following sequence: RM, MRM and RM. The MRM follows the same strategy as the RM except that, in this case, the descriptor with the largest error is replaced in each step even when the substitution does not reduce the *S* value further [6]. 

### 1.3. Stepwise Methods for the Selection of QSAR Descriptors

The forward selection method assumes no variable in Equation (1) and adds one variable at a time [9]. A regressor is inserted if it improves the model as determined by the *F*-ratio statistic. The procedure continues until no selected predictor results in an *F*-ratio value larger than a user-defined threshold. In the backward elimination method, all the variables are included in Equation (1). The predictor with the smallest *F*-ratio is deleted. The process is repeated until the deletion of any feature would produce an *F*-ratio greater than a threshold. These techniques can also be implemented to find the best *p*-term equation using criteria that are based on the residual sum of squares [9].

### 1.4. Artificial Neural Networks

Artificial neural network (ANN) models were developed [10,11], as nonlinear predictors, to calculate an output vector from the QSAR descriptors. In this work, a feed-forward ANN model with back-propagation was built to estimate the enhancement ratio. Artificial neural networks include a set of weights, *w* (to be adjusted in a training phase), bias functions *f* (.) and parameter *p*:

*y* = *f* (*x*,*w*,*p*) (2)

**Figure 1:**
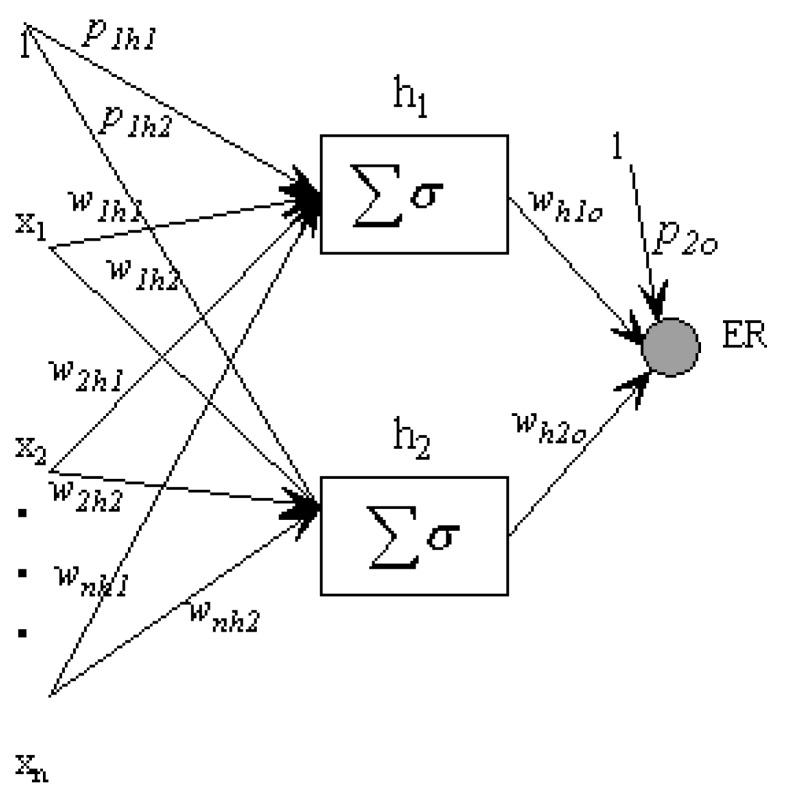
Artificial neural network model using one hidden layer and two nodes. The input vector is connected to the hidden neurons through weights *w* and *p*. The hidden layer and output variable are also connected. A nonlinear activation function σ is applied.

In a supervised ANN model, examples, representative of the process, are provided in the training phase. The best *w* and *p*, that minimize a performance criterion, are calculated. In the testing phase, new input patterns are supplied with the optimal *w* and *p* (Figure 1). Only one hidden-layer architecture is proposed for this application. There is also flexibility in the number of neurons associated with the hidden layer. Algorithms have been designed to prune large networks [12,13]. 

### 1.5. Selection of Descriptors and Prediction of ER

Three skin penetration enhancer sets were analyzed in this study. These data consist of:

- Sixty-one (61) nonpolar enhancers using hydrocortisone (HC) as a control in measuring ER (Table 1 of Iyer *et al.* [4]).
- Forty-two (42) small and relative polar enhancers using hydrocortisone (HC) as control (Table 2 of Iyer *et al.* [4] except for AT-TERPENE/HC-7 and AT-TERPENE/HC-7).
- Forty-two (42) nonpolar enhancers using Hydrocortisone Acetate (HCA) as a control (Table 3 of Iyer *et al.* [4]).

## 2. Experimental Section

The experimental method for measuring the ER was the same for all three datasets examined in this study [4]. The slope of the linear portion of the cumulative drug amount released versus the time corresponds to the steady-state flux (J, mg cm^−2^ h^−1^). The software MOLECULAR MODELING PRO™ Plus (MMP+) estimated the molecular descriptors of the 145 CPEs from their structures. MMP+ calculated approximately 114 descriptors. However, this number was reduced to 31 descriptors selected from the combined set used in studies of skin penetration enhancers provided in [4,5]. The ERM, RM and forward stepwise regression methods were introduced to discover significant relationships between a subset of structural parameters, taken from the 31 descriptors, and the ER. Statistical analyses were performed in MATLAB (The MathWorks Inc., Natick*,* MA, USA) using a QSAR/QSPR search algorithm Toolbox [6]. To minimize the possibility of correlation, the program was instructed to select eight descriptors, a number that was less than one-fifth the size of the supplied data [14]. A linear regression model was developed for each set and the following values were reported: *N*, the number of data; *R*^2^, the coefficient of determination; *S*, the standard deviation. 

## 3. Results and Discussion

The relevant descriptors obtained by the enhanced replacement method were: hydrogen bond acceptor, polar surface area, moment of inertia, glass temperature (*T*g), molar volume, radius of gyration (RG), dipole moment and polarity (Table 1). The applicability domain (AD) was not explicitly determined. However, the three data sets (*i.e.*, Database or DB #1, DB #2, DB #3) obtained from Iyer *et al.* [4] were separated on the basis of polarity and the control drug (*i.e.*, hydrocortisone and hydrocortisone acetate). In this sense, the data within a group, share a “common mechanism of action”.

The y-intercept and regression coefficients are also shown in the table. For example, the following equation was built for the 61 nonpolar enhancers (DB #1) using the ERM:

ER = −120.8055 + 80.4322 × hydrogen bond acceptor − 1.7554 × polar surface area 
− 0.0017 × moment of inertia − 0.1102 × *T_g_* − 0.0654 × molar volume +
16.9552 × *RG* − 5.0650 × dipole moment + 20.6850 × polarity (3)

**Figure 2:**
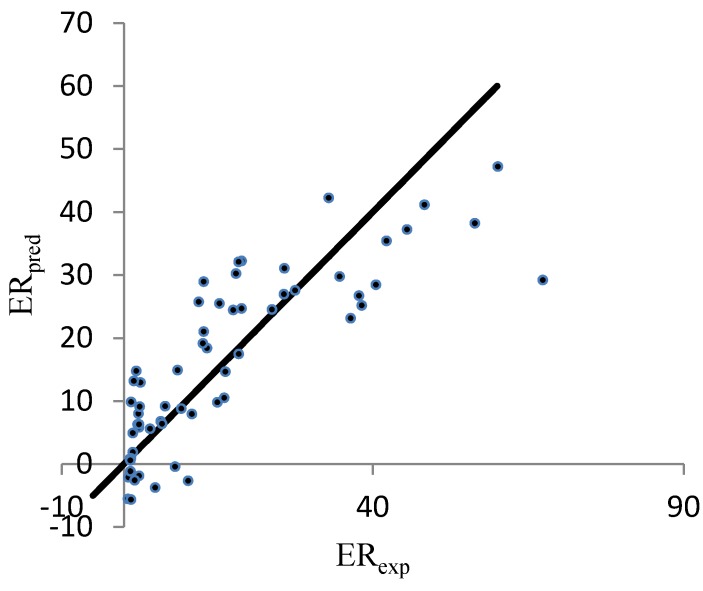
Correlation between the linear predicted and experimental ER for the set of 61 nonpolar CPEs that enhanced the permeability of hydrocortisone. The enhanced replacement method (ERM) was applied for the selection of the descriptors.

The performance of the ERM was similar to that of the RM. The stepwise method produces the lowest *R*^2^ value (*i.e.*, 0.671). Figure 2 illustrates both the experimental and predicted values.

**Figure 3:**
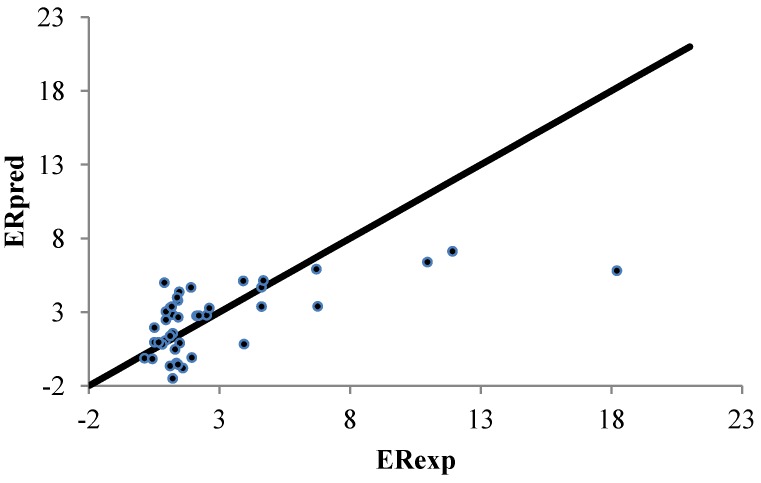
Correlation between the linear predicted and experimental ER for the set of 42 polar CPEs from DB #2. The enhanced replacement method (ERM) was applied for the selection of the descriptors.

In the work of Iyer *et al.* [4], six classic QSAR descriptors yield an *R*^2^ of 0.732. Seventy-nine classic QSAR descriptors were drawn for the analyses. A quadratic model was fitted to the data. When using the forty-two small and relative polar enhancers (DB #2), *R*^2^ values for the different methods were less than 0.4. These findings agree with the results in Iyer *et al.* [4] because no meaningful model could be developed from classic QSAR descriptors. Figure 3 shows the calculated and experimentally determined ER values for the ERM. 

**Table 1:** Results of the ERM, RM and the forward stepwise selection procedure applied to the three datasets (*i.e.*, DB #1, 2 and 3). Databases 1 and 2 were composed of 61 nonpolar and 42 polar enhancers, respectively, with hydrocortisone serving as a control molecule. Forty-two compounds were in the third database. The *R*^2^, *y*-intercepts and coefficients of the multiple linear models are listed.

	DB # 1 Nonpolar			DB # 2 Polar			DB # 3 Nonpolar		
	*N* = 61 Hydrocortisone			*N* = 42 Hydrocortisone			*N* = 42 Hydrocortisone Acetate		
	ERM	RM	Stepwise	ERM	RM	Stepwise	ERM	RM	Stepwise
*R* ^2^	0.683	0.683	0.671	0.385	0.361	0.323	0.522	0.488	0.390
Intercept	−120.81	−120.81	−132.43	−14.98	−14.50	6.39	233.64	181.96	203.21
Molecular Descriptor									
Density (g/cm^3^)									−237.02
Dipole Moment (debye)	−5.07	−5.07	−5.24						
Dispersion (J/cm^3^)^1/2^				1.57	1.02	2.76 × 10^−1^			
Number of Torsional Bonds							−12.67	−9.22	
Energy of Cohesion (J/mol)				1.00 × 10^−4^	1.00 × 10^−4^	1.00 × 10^−4^	7.00× 10^−4^		
Hydrophilic Lipophilic Balance (HLB)									
Highest Occupied Molecular Orbital (HOMO)						1.02	532.18	449.36	
Hydrogen Bond Acceptor	80.43	80.43	83.28	−3.74				127.28	92.98
Hydrogen Bond Donor					−9.81	−1.63		−121.54	
Hydrogen Bonding (J/cm^3^)^1/2^					4.36 × 10^−1^		−7.32		−5.83
Hydrophilic Surface Area (cm^2^/mol)									
Kappa 2									
Log P				−1.15					
Lowest Unoccupied Molecular Orbital (LUMO)					−7.33 × 10^−1^	−1.32			
Mean Water of Hydration									
Molar Volume (cm^3^/mol)	−6.54 × 10^−2^	−6.54 × 10^−2^							
Molecular Length (Å)			1.65				7.27	6.72	−2.56
Molecular Volumes(Å^3^)				−4.27 × 10^−1^				2.64	
Molecular Weight (Da)					−5.53× 10^−2^			−1.85	
Molecular Width (Å)				−1.51		−1.71			
Moment of Inertia (g cm^2^/mol)	−1.70 × 10^−3^	−1.70 × 10^−3^	−2.00 × 10^−3^						
Molar Refractivity (MR)							−12.10	−4.01	
Percent Hydrophilic Surface Area									
Polar Surface Area (Å^2^)	−1.76	−1.76	−1.82						−6.53× 10^−1^
Polarity (J/cm^3^)^1/2^	20.69	20.69	21.01			3.69 × 10^−1^	4.05		6.44
Radius of Gyration (Å)	16.96	16.96	12.04						
Surface Area (Å^2^)				3.39× 10^−1^			2.44		
Surface Tension (dynes/cm)				−8.79× 10^−2^	−6.90 × 10^−2^	−8.83 × 10^−2^			2.22
T_g_ (C)	−1.10× 10^−1^	−1.10 × 10^−1^	−1.01 × 10^−1^						3.83× 10^−2^
T_m_ (C)									
Water Solubility (mol/L)					2.85× 10^−1^				

**Figure 4:**
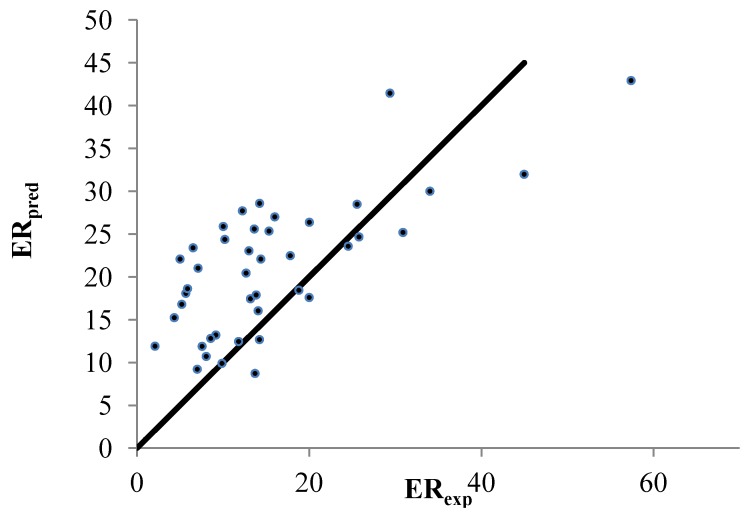
Correlation between the linear predicted and experimental ER for the set of 42 nonpolar CPEs from DB #3. The enhanced replacement method (ERM) was applied for the selection of the descriptors.

The third dataset (DB #3) yields *R*^2^ values of 0.522, 0.488 and 0.390 for the ERM, RM and stepwise method, respectively. The data are plotted in Figure 4. For the ERM, Based on 77 classic descriptors and applying a quadratic model, an *R*^2^ value of 0.738 was achieved in Iyer *et al.* [4]. It is customary to consider log(ER) instead of ER when modeling skin penetration enhancers [3]. However, this measure did not necessarily lead to improved outcomes. For example, based on the ERM algorithm, the molecular weight and Log(*P*) are determining factors in the formulation of enhancers from DB #1. Yet, a much lower *R*^2^ of 0.47 was observed. 

Replacement techniques (*i.e.*, ERM and RM) are more effective than the stepwise method in selecting the best descriptors for predicting skin penetration enhancement. Given that a multiple linear model was adopted and a relatively small pool (31 *versus* 77 and 79 features) was chosen, this approach is comparable to other techniques published in the literature. The inclusion of features, such as 4D-fingerprint descriptors [4], may help improve the performance of replacement procedures. 

**Figure 5:**
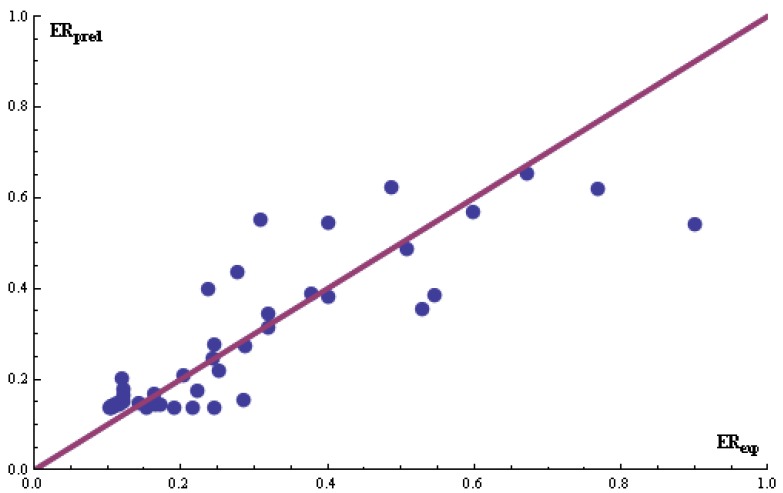
Training results for DB #1 (*N* = 47 patterns). The ERM was used for the selection of the descriptors.

The significance of the parameters to enhance drug permeation has been reported by other researchers. For example, Potts and Guy employed hydrogen bond acceptors and the molecular volume to predict skin permeability [15]. A smaller average dipole moment of the penetration enhancer causes the enhancement ratio to increase [16]. The radius of gyration, a topological index which describes the compactness of a molecule, affects the ability of the enhancer to penetrate the skin layers. This parameter was used by Zheng *et al.* [16]. In the design for skin penetration enhancers, the ERM may serve as a tool for selecting molecular structures that play a key role in promoting transdermal drug delivery. Artificial neural networks are then built to generate a more accurate model. Database #1 was selected for this application because it produced the best results. The *Neural Networks* package in Mathematica (Wolfram Reasearch, Inc., Champaign, IL, USA) was chosen for this study. The descriptors obtained by the ERM method were used as inputs to the neural networks. One hidden layer and two nodes were selected to capture the input-output relationship. A simple topology was adopted to prevent over-parameterization and to reduce the complexity of the final NN equation. The database was first randomized and divided into a training set, consisting of 75% of the compounds, and a testing set. Input and output patterns were scaled between 0.1 and 0.9. A feed-forward ANN model, composed of a sigmoid-type activation function, one hidden layer and two neurons, was constructed using eight inputs and one output (*i.e.*, an 8:2:1 network). The total number of iterations was set at 150. To avoid over-training, the training phase was instructed to stop if the MSE did not decrease steadily for a number of consecutive iterations. It took 35 iterations to identify an input-output relationship (Figure 5). The optimized neural network function, based on the scaled vectors, is:

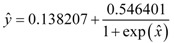
(4)
where:

*x̂* = 23.4289 − 22.7143*x̂*_1_ + 31.7314*x̂*_2_ + 32.634*x̂*_3_ + 8.81476*x̂*_4_
+ 0.274705*x̂*_5_ − 62.4769*x̂*_6_ + 20.5876*x̂*_7_ − 32.0392*x̂*_8_ (5)

and:

*ŷ* = 0.012 (*ER* − 0.63) + 0.1 *x̂*_1_ = 0.89(hydrogen bond acceptor − 0.2 ) + 0.1 *x̂*_2_ = 0.012(polar surface area − 12.03 ) + 0.1 *x̂*_3_ = 2.8×10^−5^ (moment of inertia − 732.50 ) + 0.1 *x̂*_4_ = 5.1×10^−3^ (T_g_ + 85.95 ) + 0.1 *x̂*_5_ = 2.7×10^−3^ (molar volume − 175.61 ) + 0.1 *x̂*_6_ = 0.087(RG − 3.75) + 0.1 *x̂*_7_ = 0.13(dipole moment − 0.66) + 0.1 *x̂*_8_ = 0.15(polarity − 0.47) + 0.1 (6)

**Figure 6:**
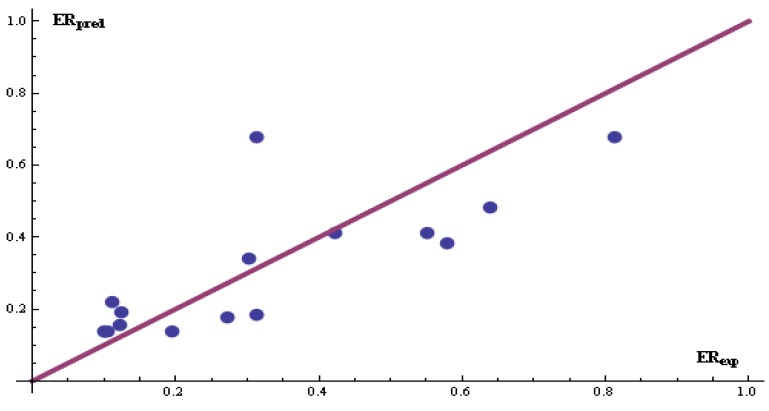
Testing results for DB #1 (*N* = 14 patterns). The enhanced replacement method was used for the selection of the descriptors.

The results of the training are shown in Figure 5 (*R*^2^ = 0.74). The ANN model outperformed Equation 1 when it is applied to the training set and was able to predict the testing set (Figure 6, *R*^2^ = 0.62).

An analytical ANN model makes it possible to design penetration enhancers based on user-defined performance criteria. The ERM-ANN approach can be extended to include the irritation potential (IP) of the CPE [5]. This strategy is suitable for estimating the flux enhancement ratio given a set of molecular descriptor values (*x*). The inverse problem [17], which consists of computing *x* necessary to meet a target ER, is more difficult. The reverse engineering of chemical structures from such descriptors is also a challenge [18]. However, robust techniques, such as the ERM, that identify the relevant features and powerful data-based modeling tools could play important roles in the process.

## 4. Conclusions

A database of skin penetration enhancers was assembled to compare the performance of replacement techniques to the more traditional stepwise method, when selecting the best molecular descriptors. The collection was divided into three sets that differ by the control compound and the polarity of the accelerants. *R*^2^ values of 0.683, 0.683 and 0.671, which corresponded to the enhanced replacement (ERM), replacement (RM) and forward stepwise methods, respectively, were computed for 61 nonpolar enhancers with hydrocortisone serving as the reference (DB #1). The other two case studies, composed of forty-two compounds each, yielded lower *R*^2^ values. Better predictions were obtained when the ERM-resulted selections for DB #1 were fed into a feed-forward back-propagation network model trained with only two hidden neurons (*R*^2^ = 0.74). The *R*^2^ value for the testing phase was found to be 0.62. Based on these results, the combined ERM and artificial neural network approach can be implemented in the design of chemical enhancers.
